# Supplementary material for: Recovery of Hydrochloric Acid from Industrial Wastewater by Diffusion Dialysis Using a Spiral-Wound Module
Source: Int J Mol Sci. 2022 Jun 1;23(11):6212. doi: 10.3390/ijms23116212 (PMC9181085; doi:10.3390/ijms23116212)
Supplement: Supplementary file 1 [file ijms-23-06212-s001.zip › ijms-1733091-supplementary.pdf]

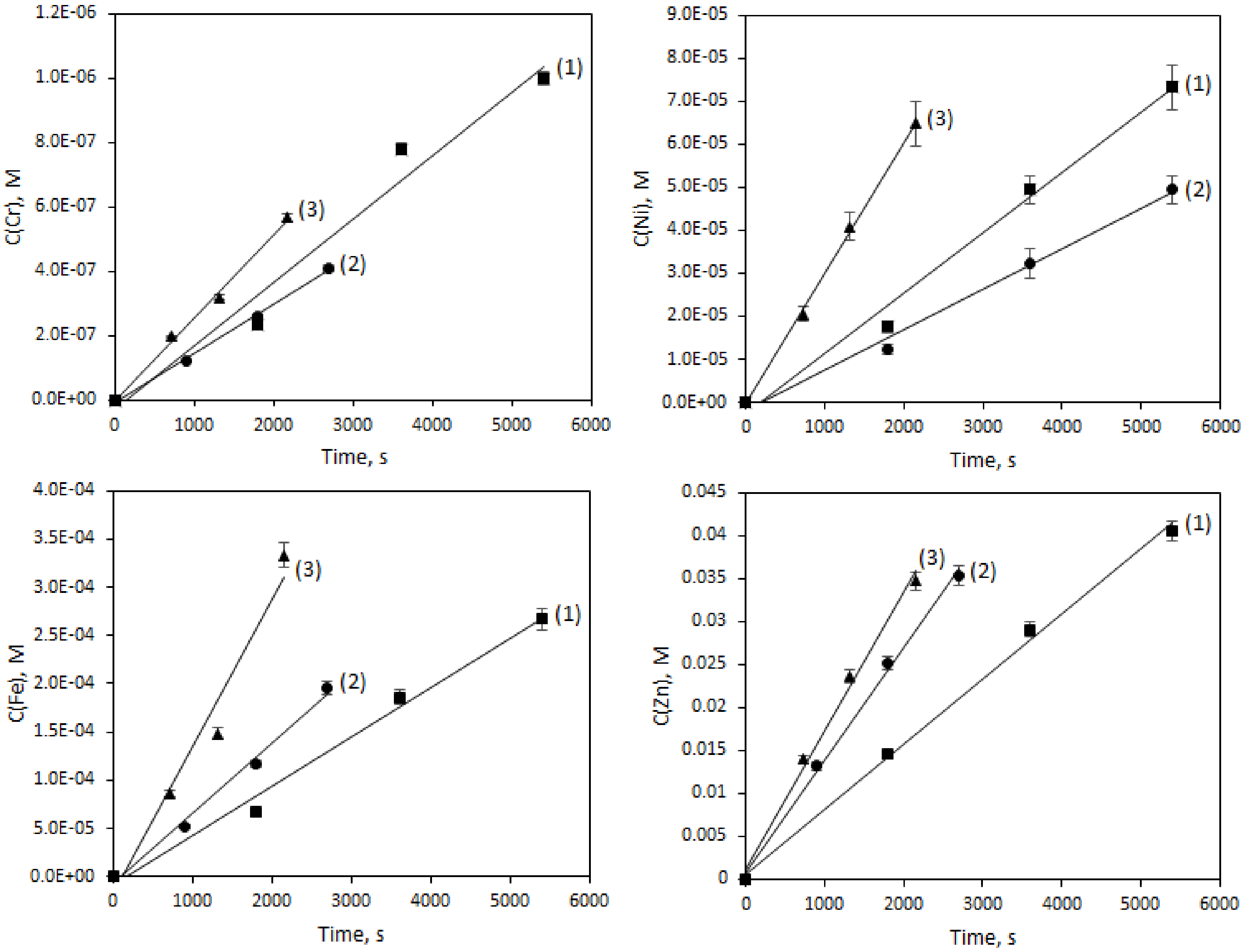

Figure S1. The concentration of metals in the dialysate chamber versus time during batch dialysis of feed solution.
